# Construction and application of a low-cost laparoscopy training simulator as a teaching tool through motor coordination and two-dimensional vision practices

**DOI:** 10.1590/0100-6991e-20223095

**Published:** 2022-02-18

**Authors:** BRENO WELLINGTON MESQUITA SILVEIRA, ARIANE LIMA DOS SANTOS, VALESKA ALVES HOLANDA, WELLYSON GONÇALVES FARIAS, ISABELE MARIA JORGE DE FREITAS, ANNYA COSTA ARAÚJO DE MACEDO GÓES, LARA BURLAMAQUI VERAS

**Affiliations:** 1 - Universidade Federal do Ceará, Departamento de Cirurgia - Fortaleza - CE - Brasil; 2 - Hospital Universitário Walter Cantídio, Departamento de Cirurgia - Fortaleza - CE - Brasil

**Keywords:** Simulation Training, Low-Cost Technology, Laparoscopy, Education, Medical, General Surgery, Treinamento por Simulação, Tecnologia de Baixo Custo, Laparoscopia, Educação Médica, Cirurgia Geral

## Abstract

**Objectives:**

to describe the construction of a low-cost laparoscopy training simulator and evaluate its level of acceptance, impact on learning, and skill development in medical students.

**Methods:**

we built a video training simulator using low-cost materials. We then carried out a cross-sectional study, with the use of an applied questionnaire to medical students.

**Results:**

51 medical students participated in the research, of whom 76.47% gained confidence in relation to laparoscopic surgery, 100% stated that the model successfully trained the skills of motor coordination and two-dimensional visual-spatial field, in addition to enabling a greater understanding of laparoscopy. All agreed that the simulator should be used before a real laparoscopic surgery scenario.

**Conclusion:**

the construction of the described laparoscopic surgery training simulator proved to be feasible and effective as an educational resource. It was well accepted by medical students, with easy handling, and promoted the development of motor and visual skills in video surgery.

## RESUMO

**Objetivos:**

descrever a construção de um simulador de treinamento de videocirurgia de baixo custo e avaliar, por meio de sua aplicação, o nível de aceitação, de impacto no aprendizado e de desenvolvimento de habilidades em acadêmicos do curso de medicina.

**Método:**

foi construído um simulador, de videotreinamento com uso de materiais de baixo custo. Realizou-se um estudo transversal com a utilização de questionário aplicado a acadêmicos do curso de medicina.

**Resultados:**

a pesquisa contou com a participação de 51 acadêmicos de medicina. Após o uso do simulador 76,47% dos pesquisados adquiriram confiança em relação à videocirurgia, 100% afirmaram que o modelo treinou com satisfação as habilidades de coordenação motora e campo visoespacial bidimensional, além de possibilitar maior compreensão da videocirurgia. Todos concordaram que o simulador deveria ser utilizado antes da situação real.

**Conclusão:**

a construção do simulador de treinamento em videocirurgia descrito mostrou-se factível e eficaz como recurso educacional. Obteve boa aceitação pelos acadêmicos, com fácil manuseio, capaz de fomentar o desenvolvimento das habilidades motoras e visuais em videocirurgia.

## INTRODUCTION

Laparoscopy is a minimally invasive surgical technique, in which the surgeon uses instruments such as tweezers, trocars, and high-definition cameras to perform a variety of procedures^1^ ^,^ ^2^. It is preferable to laparotomy due to its postoperative benefits, such as less postoperative pain, shorter hospital stay, faster return to daily activities, better aesthetic results, significant reduction in overall costs, and lower rate of infection^1^ ^-^ ^3^. 

Training simulators in laparoscopy are of great importance for the development of fundamental skills, as this modality requires the surgeon to be familiar with the use of laparoscopic equipment and to have motor coordination skills and brain adaptation to the depth perception required by using an optics with 2D vision^4^ ^-^ ^6^. Due to the importance of this training, there is a growing need for the use of simulators, in view of the positive effects on the learning curve and on the technical improvement of the practiced procedure^2^ ^-^ ^7^. 

The term “gamification” is used for the thought process and game mechanics capable of involving users in solving problem situations. It is the use of game elements or game design techniques in non-game contexts to encourage people to perform a task in a more fun way. Thus, simulations that use this concept become a potential teaching tool in the context of medical education^8^ ^,^ ^9^.

Simulation can promote learning through repetition in an environment conducive to learning, which directs the student’s attention to the step-by-step procedure^3^ ^-^ ^5^ ^,^ ^10^. Other advantages, common to other simulation models, are the ability to train without restrictions, in addition to the reduction in the use of animals^4^ ^,^ ^5^ ^,^ ^7^ ^,^ ^10^ ^,^ ^11^. However, obtaining industrialized simulators is usually costly, making this tool inaccessible and sometimes unfeasible.

 In this study, we developed a low-cost laparoscopy training model to broadly enable the use of this equipment in medical graduation, improving students’ skills and making simulation models more accessible.

The aim of this study is to describe the construction of a low-cost laparoscopy training simulator and to assess the level of acceptance, impact on learning, and development of skills in medical students, as well as repercussions in the interest in the surgical area. The main hypothesis of this study is the impact of this simulator on the development of motor and visual-spatial skills in the academic context.

## METHODS

### Construction of the Simulator

We developed two different training models, each operated inside a white plastic mannequin of male bust, 69cm x 47cm, 105 cm bust, 75cm waist and 97cm hip, acquired at the Fortaleza Equipamento Comercial, located in the city of Fortaleza, CE, Brazil. All materials used to build them were chosen with low cost and easy availability in mind.

These simulators were designed to allow more dynamic practices and to train different types of student’s motor skills. 

### Adaptation of the Mannequin

First, we made lower and upper openings to access the internal cavity. Then we installed a light bulb in the inner lower portion. The lighting wiring ran through the interior of the mannequin, passing through the upper opening, where we installed a male, bipolar, grounded, N-type plug. All parts mentioned were fixed using hot glue. Inside the mannequin, we installed a webcam close to the base, to enable the visualization of the internal cavity through external reproduction devices, such as notebooks, tablets, and the like. 

On the anterior face of the mannequin, we created three holes of 1cm in diameter, one in the umbilical region and the others in each flank, to allow the passage of the clamps to the internal cavity. 

 In the internal dorsal portion, a piece of Velcro was glued to dock the simulators (which will be described later). Finally, to occlude the opening at the bottom of the mannequin, we used a piece of wood screwed by a metal hinge. 

**Figure 1:**
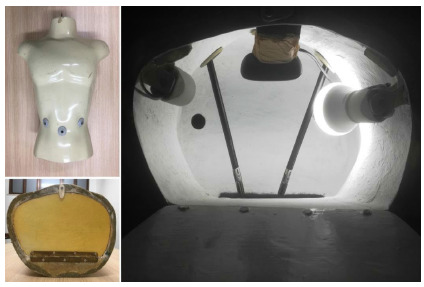
Anterior view (top left figure); base view (bottom left figure); internal view (right figure).

The surgical instrument used was a pair of Harmonic™ scalpels from the company Ethicon, Inc. The adapted mannequin is illustrated in Figure 1.

### Simulator I

**Figure 2:**
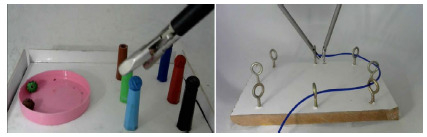
Simulator I (left figure). Simulator II (right figure).

The construction of simulator I included (Figure 2) a 15cm x 11cm x 2cm MDF (Medium Density Fiberboard) board. On the sides and top, we fixed pieces of 3mm-thick polyvinyl chloride (PVC) sheets and glued Velcro to the inferior face (to allow the simulator to be fixed to the mannequin). 

On the upper face, we glued a cylindrical container measuring 5cm in diameter and 0.4cm in height and six caps of different colored pens. Inside the container, we placed six acrylic beads (typically used in jewelry bracelets), each one of a color corresponding to a pen cap. 

The purpose of this practice is for the student to clamp the beads from the container and place each one on the respective colored cap, without allowing the bead to escape the clamp or fall onto the surface of the simulator. The task is considered complete when all six beads are in place.

In cases where the participant dropped the bead, they were instructed to prioritize the beads that were still inside the container. If there was no other bead, the instructor repositioned the dropped bead back into the container to allow the participant to retry. 

### Simulator II

The simulator II (Figure 2) was also built with a board of the same material and of the same measures as the simulator I, with the upper portion also covered with a PVC sheet and the lower portion covered with Velcro.

On the upper face, we positioned eight 33x15mm bichromated carbon steel stud-type screws, distributed in an oval fashion and equidistant from each other. It was also necessary to use some linear material that could be passed through the screw holes such as wire, probe, drain, string, or similar objects. In this study, we opted for a silicone nasogastric catheter that was painted blue. 

The purpose of this practice is for the student to pass the catheter through all the holes in the studs.

### Study Design

We carried out a cross-sectional study to evaluate this low-cost model of training in laparoscopy developed by medical students.

The invitation to participate in the study was disclosed online through a Google Forms^®^ form. In this form, we obtained information on identification (name, sex, age, college, and medical school semester), contact (email and phone number), and previous experience with the subject, whether practical, simulated, or theoretical. 

The criterium for inclusion in the study was being a medical student, regardless of institution, semester, and previous experience with laparoscopy. The exclusion criterium was not having shown up at the study location on the scheduled day. 

The research sample was a convenience one, initially counting on the enrollment of sixty-nine undergraduates, corresponding to the tolerated limit of participants in the environment. Participants were summoned and evenly distributed on different days of the week to avoid crowding. Eighteen participants abandoned the research before starting the next steps, as they did not show up at the research site on the informed day and time, resulting in a final number of 51 participants.

Initially, students went to the room where they filled out the Informed Consent Form (FICF) and the pre-simulation questionnaire that assessed their level of safety in relation to the procedure (little, medium or high). 

Then, participants watched a short lecture on laparoscopic surgery, which focused on key concepts and fundamentals, as well as on instruments manipulation. After this moment, the students were directed to the simulation room, where the two simulators were, as well as two instructors, each responsible for one simulator. Before the start of the simulation, the instructors presented the simulator and the tweezers to the participants, highlighting the correct handling of the equipment. They also passed on instructions about the task to be performed, the deadline for completion, and informed that, during the simulation, participants would be submitted to an evaluation through a checklist. 

The practice time in each simulator was five minutes. The simulation was considered finished when the student completed the task or when the stipulated deadline ended.

For each simulator there was a specific checklist. However, both had in common the evaluation of the use of both upper limbs, the rotational joint, and the simulation completion time. The specific points of simulator I referred to the number of beads positioned at the end of the simulation and the total number of attempts made, while those of simulator II referred to the number of holes passed through by the catheter at the end of the simulation.

After the end of the activity, participants answered a post-test questionnaire, which returned to the question of the level of safety made in the pre-test and evaluated eight other aspects through the following questions: (I) Do the mobility and instrument introduction resemble reality? (II) Does the simulator allow training in motor coordination? (III) Does the simulator train the brain for 2D perception? (IV) Is the simulator easy to use? (V) Does the simulation motivate the study of laparoscopy? (VI) Does the simulator enable a better understanding of laparoscopy? (VII) Does the simulator consolidate learning? (VIII) Should the simulator be used as a training method before the real situation? 

Each question had the following alternatives as a possibility: totally agree (TA), partially agree (PA), indifferent (I), partially disagree (PD), and totally disagree (TD). At the end of the post-test questionnaire, we also asked the student’s perception of the stage of greatest difficulty for each simulator. This study was submitted and approved by the Ethics in Research Committee of the Federal University of Ceará (CEP/UFC/PROPESQ) under protocol number 4.143.343.

### Statistical Analysis

We tabulated the collected data in Microsoft Excel^®^ spreadsheets and evaluated them using the statistical software GraphPad Prism9® with the t test for paired samples. The rejection of the null hypothesis was established for values of p<5%. 

To calculate the sample, we used the G-Power 3.1.9.2 software, with which we found the following: sample power of 0.8, significance level 0.05, and effect size 0.5. The minimum sample found was 35 participants, and we included a total of 51 students. We assessed the difference between the students’ responses with the Wilcoxon W test and estimated the normality of statistical data with the Shapiro-Wilk test (0.8, p<0.001).

## RESULTS

The research comprised 51 medical school students, 25 of them female and 26 male. The age of respondents ranged between 18 and 41 years for females, with an average of 23.6 years, and between 18 and 38 for males, with an average of 23.6 years. Research participants were students attending between the 1st and 9th semester, with the majority (56.8%) between the 1^st^ and 4^th^ semester. In addition, 15.7% had previous experience with the use of simulators and 7.8% had real practical experience.

In the question “Do you feel safe to undergo any video-surgical procedure?”, 50 out of 51 respondents (98.0%) initially stated that they were unsafe. After the simulation, 39 out of 51 (76.4%) claimed to have acquired some degree of confidence in performing the procedure (p<0.0001) and 6 out of 12 (50.0%) who claimed to be insecure after the practice had a reduction in their level of insecurity. These data are illustrated in Table 1.

**Table 1:** Safety for performing laparoscopy in a real situation.

	No			Yes		
	GINS	MINS	LINS	LSEG	MSEG	GSEG
Before Simulation	76.4	17.6%	3.9%	0%	1.9%	0%
After Simulation	9.8%	11.7%	1.9%	43.1%	33.3%	0%

GINS: Great insecurity. MINS: Medium insecurity. LINS: Little insecurity. LSEG: Little security. MSEG: Medium security. GSEG: Great security.

In the post-test, 48 participants stated that the introduction of tweezers and their mobility simulated reality well, 50 stated that the simulation motivated the study of laparoscopy, and all stated that the simulation trained motor coordination and the brain for 2D perception, allowed a better understanding of laparoscopy, consolidated learning, and should be used as a training method before the real situation (Table 2).

**Table 2:** Post-test results.

ASPECT	TA	PA	I	PD	TD
Does the introduction of instruments and mobility simulate reality well?	52.9%	41.1%	3.9%	1.9%	0%
Does the simulator allow you to train motor coordination?	92.1%	7.8%	0%	0%	0%
Does the simulator train the brain to 2D perception?	92.2%	7.8%	0%	0%	0%
Is the simulator easy to use?	49.0%	37.2%	3.8%	9.0%	0%
Does simulation motivate the study of laparoscopy?	92.2%	5.8%	0%	1.9%	0%
Does the simulator enable a better understanding of laparoscopy?	90.2%	9.8%	0%	0%	0%
Does the simulator consolidate learning?	86.3%	13.7%	0%	0%	0%
Should the simulator be used as a training method before the real situation?	98.04%	1.96%	0%	0%	0%

*TA: totally agree. PA: partially agree. I: indifferent. PD: partially disagree. TD: totally disagree.*

Regarding the simulation checklist for simulator I, 40 participants used both limbs and 34 used the rotational pinch joint. The average number of beads placed on the caps per participant was 3.68. The average number of bead positioning attempts per participant was 8.8. Six participants were able to complete the simulation within the stipulated time, with an average time of 3 minutes and 55 seconds. Regarding the use of simulator II, 50 students used both limbs and 29 used the rotational pinch joint. The average number of passed through holes was 3.98 per participant. Four managed to complete the simulation within the stipulated time, with an average time of 4 minutes and 16 seconds.

We asked which stage of the simulation was more difficult to practice in each simulator, and more than one option could be selected. The results are shown in Table 3.

**Table 3:** Major difficulties faced by participants when using simulators I and II.

Simulator I				
CB	KBCC	PBC	DP	UTRJ
18 (35.3%)	3 (5.3%)	16 (31.4%)	26 (50.9%)	9 (17.6%)
Simulator II				
CC	KCCC	2T	DP	UTRJ
5 (9.8%)	7 (13.7%)	12 (23.5%)	38 (74.5%)	10 (19.6%)

CB: clamp bead. KBCC: keep beads under clamp control. PBC: place beads in the caps. DP: depth perception. UTRJ: use tweezer rotational joint. CC: clamp the catheter. KCCC: keep catheter under clamp control. 2T: need to use 2 tweezers.

## DISCUSSION

Laparoscopy is a surgical modality of enormous importance, being chosen as the method of choice in several situations. Despite this relevance, there is little practical contact with laparoscopy by medical students, and this lack of experience leaves the student insecure because they are not familiar with the equipment or with the basic knowledge of instrument manipulation. 

The simulator was built in a simple way and with cheap materials, allowing an easy reproduction. Its use and assembly are practical, and it can be used over and over again. 

The laparoscopy model uses the “gamification” method, which makes the learners the protagonists of their own learning, with a playful approach through simple tasks, in a controlled environment, with goals to be accomplished each time. Such an approach was able to motivate the student, stimulate cognitive skills, adaptation, and creativity, which was achieved in a more pleasant way, avoiding situations of pressure, stress, or anxiety^8^ ^,^ ^9^. 

The simulator aims to associate theoretical content acquired in the classroom during graduation with practice, establishing fundamental concepts and training manual skills that would hardly be developed with theory alone^1^ ^,^ ^12^. This teaching method with the use of simulators is widespread in surgical residency programs, to make residents fit before performing surgical procedures on patients^1^ ^,^ ^5^ ^,^ ^13^. Model learning is divided into three parts: the rapid acquisition of manual skills, the consolidation of learning, and the retention of skills. The more you practice, the greater the improvement in the learning curve until plateau is reached^5^ ^,^ ^13^ ^,^ ^14^. 

It is noteworthy that the intention of the simulator described in the article is not to train robust or complex surgical procedures, but to direct primary learning to an unfamiliar audience, especially with regard to the manipulation of instruments and main movements, adaptation to the 2D visual-spatial field, and motor coordination practices^6^ ^,^ ^15^. We should also mention that the simulated training of surgical skills improves manual skills and protects the learner and the patient due to the controlled environment, reducing the risk of iatrogenic episodes^2^ ^,^ ^11^ ^,^ ^13^ ^,^ ^14^ ^,^ ^16^ ^,^ ^17^. 

Based on the questionnaires applied, the use of the simulator enabled students to handle surgical instruments and to understand the visual-spatial field, in addition to consolidating theoretical learning and stimulating the study of laparoscopy. Thus, in agreement with the literature, the use of the simulator was able to increase students’ safety regarding laparoscopy and was judged by them as a method that should be used before any real situation^6^ ^,^ ^13^ ^,^ ^14^ ^,^ ^17^.

The model built, in addition to the low cost, was advantageous in preparing students for their first contact with laparoscopy, stimulating studies in this area and making them more prepared for a real future experience, as well as allowing greater dissemination of this surgical modality and enabling more complete academic training. Thus, it is possible to affirm that low-cost simulators, when well-planned, can serve as alternatives to high-cost industrial simulators, making this teaching tool more accessible to the learning public. 

Few students had previous experience with laparoscopy, 2 out of the 51 participants. Due to discrepancy between the groups, no comparisons were made, as there was not enough to allow a conclusion with an acceptable level of significance. 

The limitations of this study are simulation time, inappropriate material, previous experience in laparoscopy, and use only by students. The number of participants was based on entries per digital platform. The simulation time had to be limited by logistical reasons, as the students’ inability could prolong the completion of the simulated task indefinitely. Appropriate laparoscopic instruments (such as needle holders or grasping forceps) were not used due to high cost, which would not meet the objective of the study. 

New studies should be carried out for more reliable results, with surgical residents and specialist physicians, experienced in performing laparoscopy on real patients, to confirm that the use of the simulator resembles the real situation and contributes to the introduction of students still inexperienced in the practice of laparoscopy. Also, simulations with longer time and more realistic tasks, such as ligation of structures like artificial vessels and appendages, will make the simulation even more complete^3^.

## CONCLUSION

The low-cost laparoscopy training simulator was well accepted by medical students and proved to be feasible and effective as an educational resource, promoting the development of motor and visual skills in laparoscopy, reducing insecurities and fostering interest towards the surgical area.
